# Pseudomelanosis Duodeni of Undetermined Etiology

**DOI:** 10.4021/gr465w

**Published:** 2012-07-20

**Authors:** Samit S. Jain, Dharmesh K. Shah, Amol A. Khot, Narendran R. T, Amit R. Gharat, Pravin M. Rathi

**Affiliations:** a Department of Gastroenterology, T.N.M.C , B.Y.L, Nair Hospital, Mumbai, India

**Keywords:** Duodenum, Pseudomelanosis duodeni, Pigmentation, Iron, Melanin

## Abstract

Pseudomelanosis duodeni is a rare, benign condition of unknown etiology. It is characterized by collection of pigment-laden macrophages in the tips of duodenal villi. The pigment, originally interpreted as melanin, pseudomelanin, lipomelanin or hemosiderin, has now been demonstrated to be mostly ferrous sulfide. There is a strong association with chronic renal failure, arterial hypertension, diabetes mellitus and the use of medications such as ferrous sulfate, hydralazine, propranolol, hydrochlorothiazide and furosemide. We reported a case of a 48 years old female who only had dyspeptic symptoms and no history of hypertension or drug history. Laboratory tests showed normal serum electrolytes and renal function. On endoscopy we found multiple tiny brownish-black pigmentation throughout proximal duodenum. Histopathological examination showed mild inflammation in lamina propria with haemosiderin-laden macrophages. Stain for iron was positive and that for melanin was negative.

## Introduction

Pseudomelanosis duodeni is a rare, benign condition of unknown etiology. It is characterized by collection of pigment-laden macrophages in the tips of duodenal villi. Several case reports and a few case series have been described in the literature. In most cases, risk factors were found like oral iron intake, hypertension, chronic renal failure, drugs like furosemide, hydralazine, propranolol, but in our case, patient diagnosed having pseudomelanosis duodeni, had just dyspeptic symptoms and didn’t have any risk factors.

## Case Report

**Figure 1:**
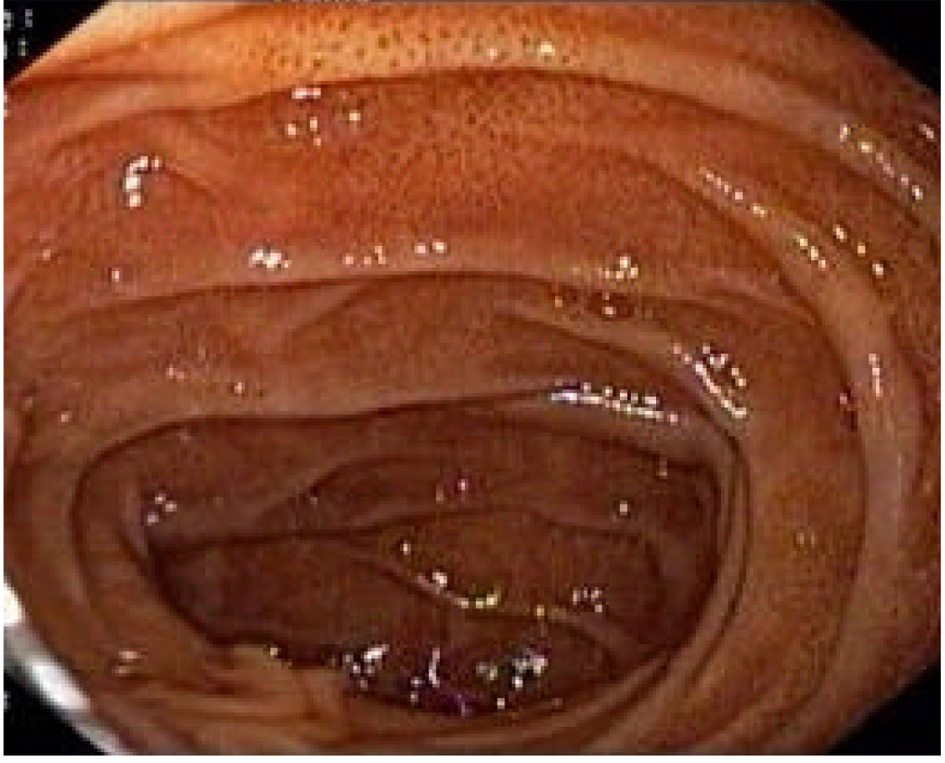
Esophagogastroduodenoscopy showing muliple dark pigmented spots in the second portion of the duodenum.

**Figure 2:**
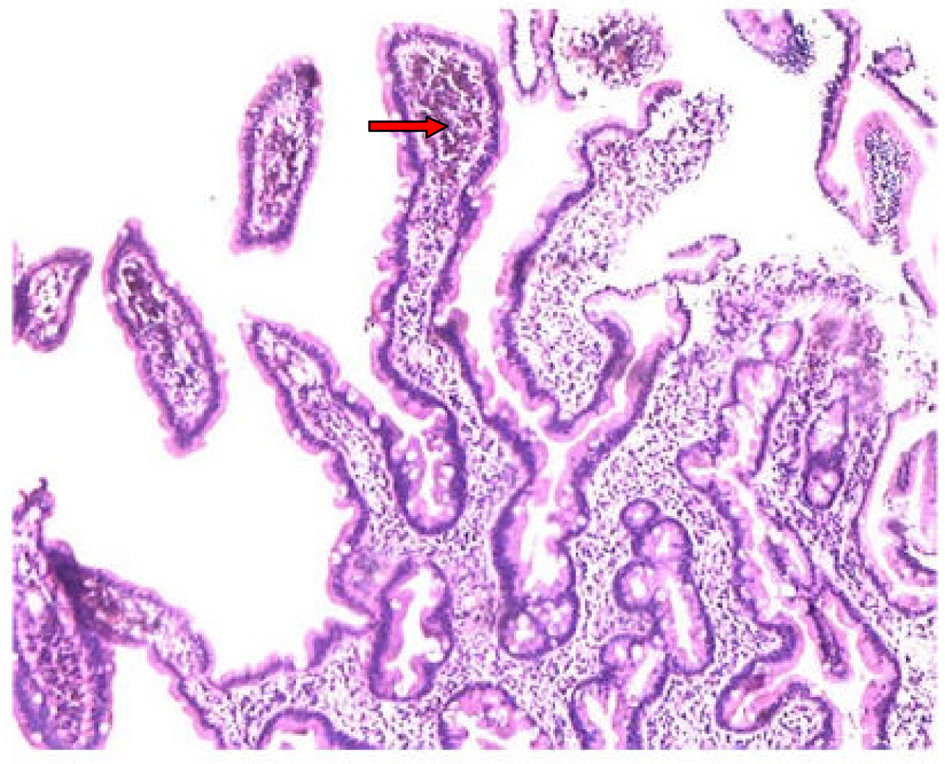
Histological examination of a duodenal biopsy showing pigmented material (red arrow) deposited in the macrophages of the lamina propria.

**Figure 3:**
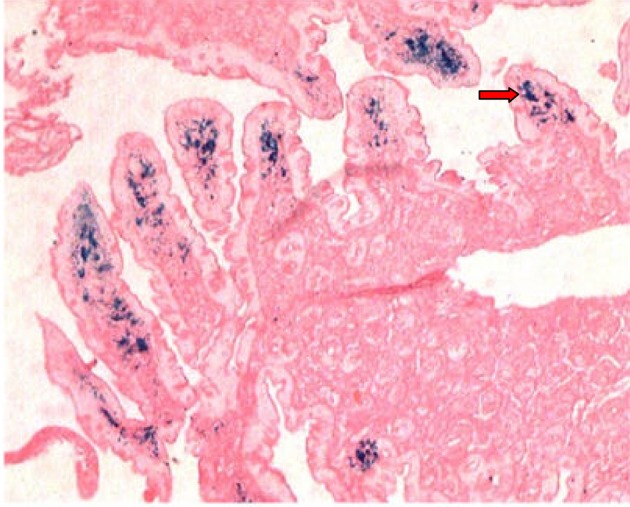
Positive Perls Prussian blue stain of duodenal biopsy showing blue color pigmentation (red arrow).

A 48 years old female presented to our hospital with the complaint of epigastric pain since 10 months which was constant, dull aching, non-radiating and occasionally aggravated after meals. There was no history of vomiting, regurgitation, abdominal distension, hematemesis, malena or abdominal lump. There was no history of loss of appetite or weight loss. For above symptoms, patient had tried proton pump inhibitors intermittently for one month, but there was no improvement. Laboratory tests showed normal hemogram, serum electrolytes and renal function. Ultrasonography was also normal. On performing upper gastrointestinal endoscopy we found normal esophagus, mild antral erythema, along with multiple tiny brownish-black pigmentation throughout proximal duodenum (first and second part of duodenum) (Fig. 1). Antral and duodenal biopsies were taken. Histopathological examination of antral biopsy showed mild antral gastritis with no evidence of helicobacterium pylori infection and duodenal biopsy showed mild inflammation in lamina propria with haemosiderin-laden macrophages (Fig. 2). Perls prussian blue stain for iron was strongly positive (Fig. 3) and Masson-Fontana stain for melanin was negative. A diagnosis of pseudomelanosis duodeni was confirmed. This case has been reported since there were no risk factors associated with pseudomelanosis duodeni as described in previous case reports and case series.

## Discussion

Pseudomelanosis duodeni is a rare benign condition first described by Bisordi and Kleinman in 1976 as “melanosis duodeni”, since the real nature of the black-brown pigmentation of the mucosa had not been further investigated [1]. The pigment, originally interpreted as melanin [1, 2], pseudomelanin, lipomelanin or hemosiderin, has now been demonstrated to be mostly ferrous sulfide [3] with small amounts of other elements. It is characterized by collection of pigment-laden macrophages in the tips of duodenal villi. Several case reports and a few case series have been described in the literature. The largest case series included 17 adult patients [4].

Pseudomelanosis duodeni occurs predominantly in middle-aged to old adults and more commonly in females (1.2 - 2:1) [4]. There is a strong association with chronic renal failure, arterial hypertension, diabetes mellitus and the use of medications such as ferrous sulfate, hydralazine, propranolol, hydrochlorothiazide and furosemide [5]. Some cases have been associated with gastrointestinal bleeding.

Pseudomelanosis duodeni per se doesn’t cause any symptoms. Most of the time, it is the incidental finding on endoscopy, which makes the diagnosis, as it was in our case. The source and pathogenesis of pigment deposition exclusively in the duodenal mucosa and the prognostic implications of pseudomelanosis duodeni are unknown. Unlike iron or other heavy metal deposits elsewhere in the body, which may generate a fibro inflammatory reaction, there has been no documented fibrosis, duodenitis, stricture formation or ulceration described in the literature.

At upper endoscopy the duodenum mucosa is speckled with multiple discrete, flat, small dark spots, mainly in the proximal portions (bulb and second portion) [2]. Occasionally the brownish black spots may be seen in the mucosa of stomach, jejunum or ileum [6]. Muliple mucosal biopsies are required for diagnosis. A collection of macrophages packed with a characteristic brown-black granular pigment is seen within the lamina propria of the villi on routine optical microscopy. Black-brown pigment may also be seen in epithelial cells or extracellular matrix [2]. Histochemichal stains for iron (Perl’s prussian blue) or melanin (Masson-Fontana) may be positive, but are unpredictable [5].

Though microscopic diagnosis of pseudomelanosis duodeni is quite straightforward, its endoscopic and histopathological differential diagnoses include metastatic melanoma and other pigmentations including exogenous ingested substances like charcoal [7].

The diagnostic and prognostic significance of pseudomelanosis duodenal has yet to be determined and the appropriate follow-up, if any, is unclear. No specific therapeutic or follow-up protocol is recommended [4].
